# High-power, short-duration ablation in the coronary sinus: clinical cases and preliminary observations on swine hearts

**DOI:** 10.1007/s10840-021-00994-0

**Published:** 2021-04-15

**Authors:** Chengming Ma, Xiaomeng Yin, Yunlong Xia, Jiao Sun, Shiyu Dai, Lianjun Gao, Xianjie Xiao, Yuanjun Sun, Rongfeng Zhang, Yingxue Dong, Zhongzhen Wang, Xiaohong Yu

**Affiliations:** 1 Department of Cardiology, Institute of Cardiovascular Diseases, First Affiliated Hospital of Dalian Medical University, 193# Lianhe Road, Shahekou District, Dalian, 116011 China; 2 Department of Graduate school, Dalian Medical University, Dalian, China

**Keywords:** High-power and short-duration, Radiofrequency ablation, Coronary sinus, Atrial fibrillation

## Abstract

**Purpose:**

Coronary sinus-related arrhythmias are common; however, it is difficult to perform radiofrequency (RF) ablation at these sites efficiently and safely. High-power, short-duration ablation (HPSD) is a proven alternative strategy for pulmonary vein isolation (PVI); whether it can be applied to ablation of the coronary sinus is unknown. The purpose of this preliminary study was to evaluate the feasibility and safety of HPSD ablation in the coronary sinus.

**Methods:**

Firstly, we demonstrated 4 clinical cases of 3 types of arrhythmias who had unsuccessful ablation with standard power initially, but received successful ablations with HPSD. Secondly, RF ablation was performed in the coronary sinus ostium (CSO) and middle cardiac vein (MCV) of 4 *in vitro* swine hearts. Two protocols were compared: HPSD (45 W/5 S×5 rounds) and a conventional strategy that used low-power, long-duration ablation (LPLD: 25 W/10 S ×5 rounds). The total duration of HPSD protocol was 25 s, and which of LPLD was 50 s.

**Results:**

A total of 28 lesions were created. HPSD can produce longer, wider, deeper, and larger lesions than LPLD. This difference was more pronounced when the ablation was in the MCV. One instance of steam pop occurred during LPLD in the MCV.

**Conclusions:**

HPSD is an effective alternative strategy for ablation in coronary sinus according to clinical applications and preliminary animal study. However, the safety needs to be further evaluated based on more animal and clinical studies.

## Introduction

Coronary sinus-related arrhythmias are common; however, it is difficult to perform radiofrequency (RF) ablation at these sites efficiently and safely due to their anatomical and electrophysiological characteristics. High-power, short-duration ablation (HPSD) was originally used for pulmonary vein isolation (PVI); it improves the relationship between resistive heating and conductive heating and has been proven to be an alternative strategy. Whether it can be applied to ablation in the coronary sinus is unknown. Recently, we have performed HPSD in the coronary sinus successfully in 4 patients who had unsuccessful treatment with standard power. Then, we investigated the effect of HPSD on lesion creation in the coronary sinus of a swine model.

## Methods

The inspiration for this study design was derived from 4 patients with coronary sinus-related arrhythmias who had unsuccessful ablation with standard power initially, but ultimately received successful ablation under HPSD in our center. The patients provided informed consent for the publication of their cases. The 2 strategies, LPLD followed by HPSD, for each patient were performed by the same operator.

The experimental study showed relevant data to compare the geometric parameters of HPSD lesions and conventional low-power, long-duration ablation (LPLD) lesions in different coronary sinus locations (middle cardiac vein (MCV) and coronary sinus ostium (CSO)). The Institutional Ethical Committee for animal research approved the experimental protocol.

### Case reports

#### Case 1

**Fig. 1:**
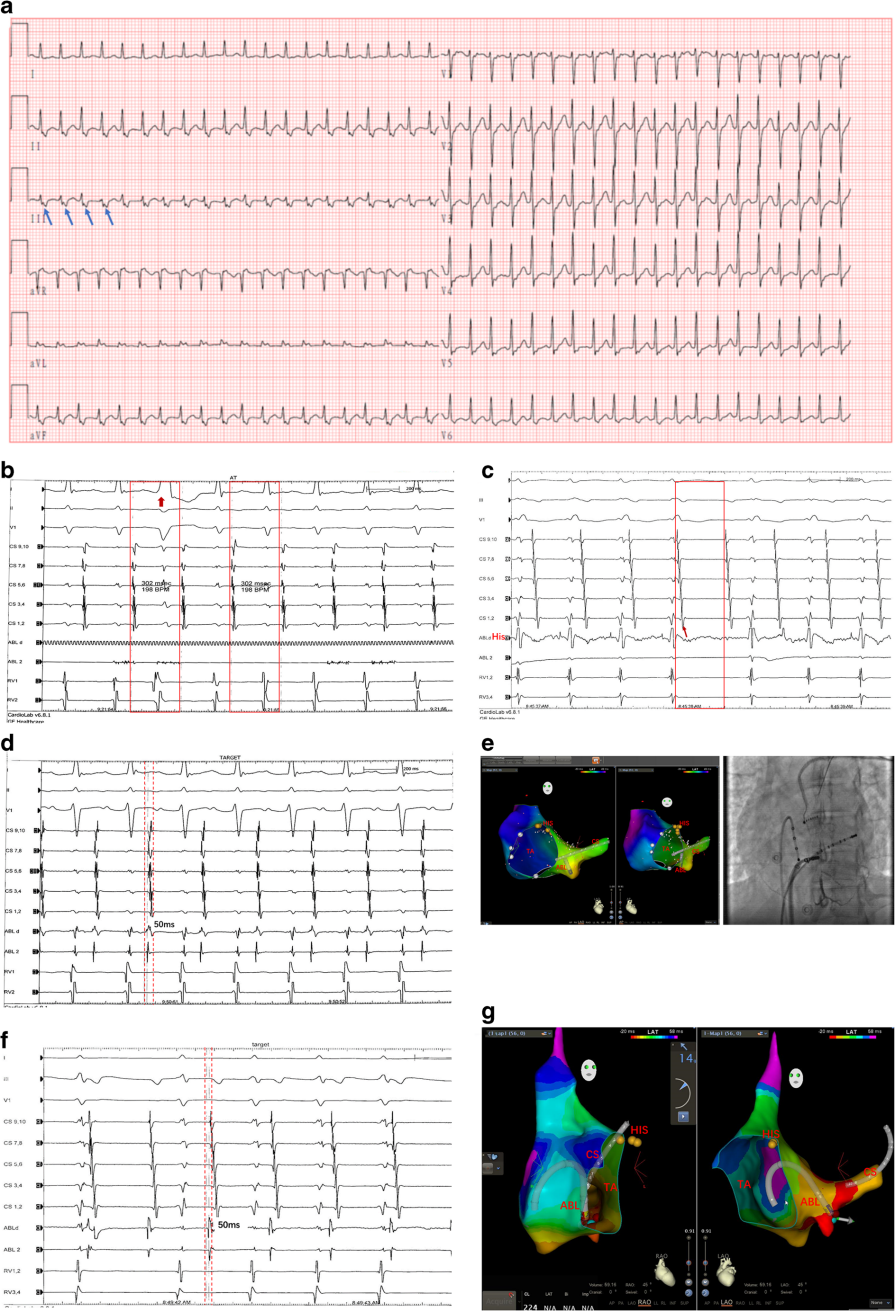
Case 1-Surface ECG and intracardiac electrogram during EPS study**. a** A supraventricular tachycardia, P wave (blue arrow) can be detected on end of each narrow QRS complex. **b** A premature ventricular excitation (red arrow) did not change tachycardia. **c**, Wenckebach’s conduction was founded during tachycardia. Note, the fourth A wave did not conduct to ventricle. **d** Shows the ablation site. **e** Left panel shows LAO and anteroposterior view in 3D electroanatomic mapping system (Carto3, Biosense Webster, Diamond Bar, Calif) of ablation site in MCV. Right panel shows coronary venography. **f**,**g,** In second procedure, ABL catheter was placed in MCV and obtained satisfactory ablation target which was same as first procedure. (His, the site of His potential; CS, coronary sinus catheter; ABL, ablation catheter; TA, tricuspid annulus)

A 25-year-old female patient with recurrent palpitations for almost 3 months was diagnosed with atrial tachycardia (AT) in our center. She had no history of structural heart disease. The electrocardiogram (ECG) in the emergency room showed tachycardia with narrow QRS complexes, and her heart rate was 241 beats per minute (Fig. 1a). The RR intervals were consistent, and a P wave that was negative in the II, III, and aVF leads and positive in the aVL, aVR, and V1 leads was observed at the end of each QRS complex. Intravenous injection of verapamil did not resolve it. Transthoracic echocardiography found a left ventricular ejection fraction (LVEF) of 35%.

A narrow QRS tachycardia with a cycle length (CL) of 302 ms was induced with programmed stimulation in the atrium during an electrophysiological study (EPS), and it was not influenced by premature ventricular excitation (Fig. 1b). Furthermore, Wenckebach’s phenomenon of conduction between the atrium and ventricle was detected for some time, confirming this tachycardia as AT (Fig. 1c). The earliest atrial potential (AP) was observed at CS7,8 and 50 ms preceding the onset of the P wave on surface ECG. We placed an irrigated-tip radiofrequency catheter (Thermocool SMART-TOUCH CF-sensing ablation catheter, Biosense Webster, Diamond Bar, Calif) via the femoral vein approach, and a combination of atrial and ventricular potential (VP) and earliest activation was mapped in the MCV (Fig. 1d). Coronary angiography and venography were performed to locate the MCV and assure a safe proximity between it and the adjacent coronary artery (Fig. 1e). RF energy was applied at a maximum power of 20 W with an irrigation rate of 60 mL/min and temperature of 50 °C, and the tachycardia disappeared after 2 s of application. The application was discontinued once a rise in impedance was noted, and the total application time was 120 s. No tachycardia could be induced by programmed atrial stimulation or following an intravenous drip of isoprenaline. Then, the procedure ended.

Unfortunately, she suffered from AT again 5 hours post-procedure, and the ECG demonstrated the same manifestation as that present pre-procedure. The acute recurrence might be due to inadequate energy delivery in the vessels, and an EPS was carried out again the following day. As suggested by the previous and second EPSs, a satisfactory ablation target was mapped at the same MCV site as in the previous procedure (Fig. 1f, g). We tried the HPSD strategy (45 W/5 S) with an irrigation rate of 30 mL/min and a 56-hole irrigated-tip RF catheter (Thermocool Smarttouch SF; Biosense Webster, Diamond Bar, Calif), and the tachycardia disappeared once energy was applied. The tachycardia terminated successfully after a total of 60-s application. No chest pain occurred during energy delivery, and no coronary artery was damaged according to coronary angiography. No tachycardia occurred during the 6-month follow-up, and transthoracic echocardiography demonstrated that the LVEF recovered normally.

#### Case 2

**Fig. 2:**
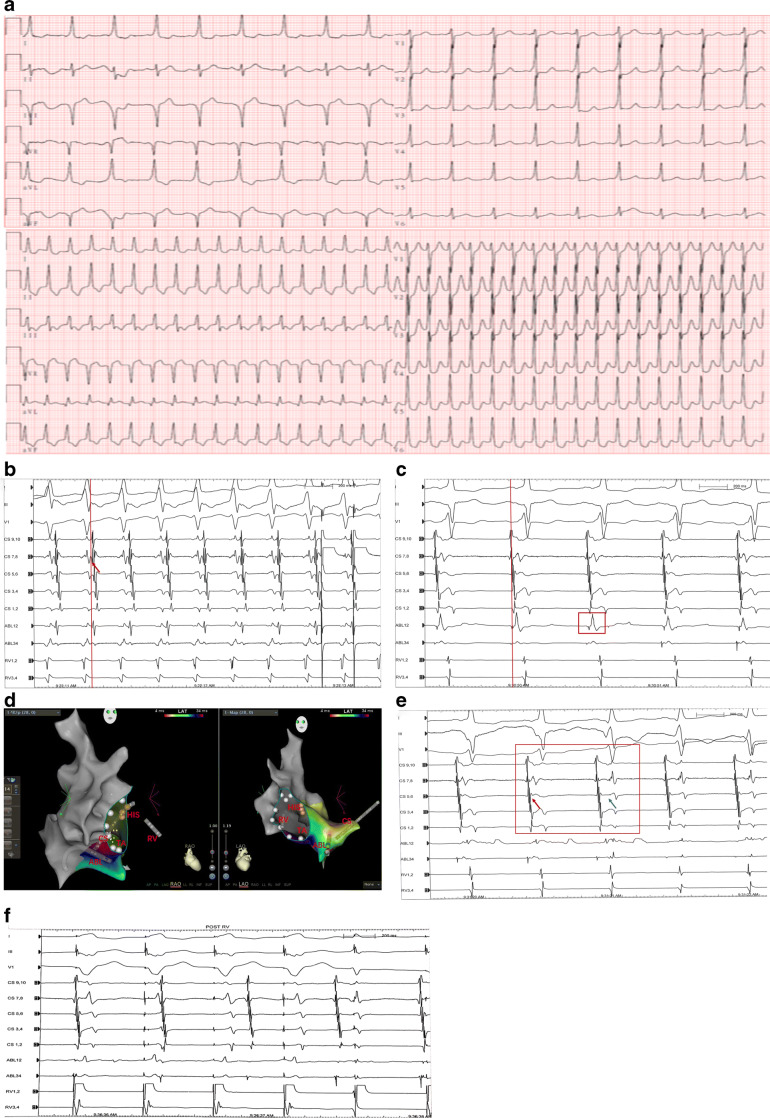
Case 2-Surface ECG and intracardiac electrogram during EPS study**. a** The upper figure shows pre-excitation of ventricle in sinus rhythm. The below figure demonstrates the tachycardia. **b** The earliest retrograde AP was detected in CS7,8 during tachycardia. **c** ABL catheter recorded the earliest antegrade ventricular activation (vertical red line) in sinus rhythm and a combined a-V electrogram (red box). **d** Shows the ablation site. **e** Ablation at MCV terminates pre-excitation of ventricle successfully (red box). The fusion of AV potential (red arrow) dissociated (blue arrow) during ablation. **f** VA retrograde conduction was terminated

A 28-year-old female patient suffered from palpitations for almost 10 years, and her symptoms worsened within 3 months. The ECG in sinus rhythm demonstrated pre-excitation syndrome located in the posterior septum pathway, and tachycardia with a narrow QRS complex is shown in Fig. 2a. The QRS complex in leads II, III, and aVF manifested a “QS” pattern with a negative δ wave, and that in lead V1 manifested a “rS” pattern with a positive δ wave. The tachycardia could be terminated by administration of amiodarone or verapamil. She was diagnosed with Wolff-Parkinson-White (WPW) syndrome and admitted to our center. A normal LVEF was found in transthoracic echocardiography, and no structural heart disease was observed.

During an EPS, when pacing at the right ventricular apex, the ventricle-atrium (VA) 1:1 retrograde conduction was centripetal, and the earliest AP was detected in the proximal CS at CS7,8. Decremental VA conduction was not observed. While the CL of atrial S1S1 and S1S2 programmed stimulation decreased, the pre-excitation of the ventricle increased. Atrial stimulation with a CL of 260 ms induced tachycardia with a narrow QRS complex, which was consistent with clinical ECG. The earliest AP was observed at CS7,8 during tachycardia (Fig. 2b) and orthodromic atrioventricular reentrant tachycardia due to the posterior accessory pathway was confirmed. Mapping was performed on the mitral annulus via atrial septal puncture, and the left accessory pathway was excluded. The RF catheter (Thermocool SMART-TOUCH CF-sensing ablation catheter, Biosense Webster, Diamond Bar, Calif) was inserted into the coronary sinus, and a combined atrial-Ventricular (a-V) electrogram was obtained in MCV (Fig. 2c, d). The ventricular potential was 30 ms preceding the onset of the δ wave on ECG. RF ablation was performed with a setting of 35 W/50°C, but VA retrograde conduction could not be blocked. Then we tried HPSD strategy. A 56-hole irrigated-tip RF catheter (Thermocool Smarttouch SF; Biosense Webster, Diamond Bar, Calif) was positioned at the same site in MCV and VA retrograde conduction was easily blocked within 2 s following ablation at this site (Fig. 2e). RF energy was applied under the following: 45 W/5 S with an irrigation rate of 30 mL/min and a total RF time of 50 s. After that, ventricular pacing showed VA retrograde conduction disconnected (Fig. 2f), and no tachycardia could be induced by programmed stimulation or an intravenous drip of isoprenaline. No tachycardia occurred during the 12-month follow-up, and no ventricular pre-excitation was detected in sinus rhythm.

#### Case 3

**Fig. 3:**
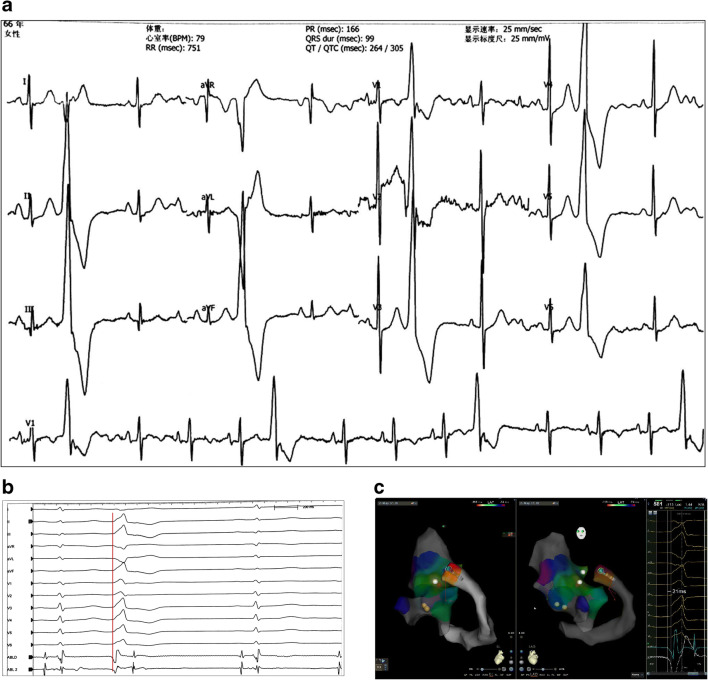
Case 3—surface ECG and intracardiac electrogram during EPS study**. a** The ECG demonstrated PVC. **b**, **c** Shows the ablation site

A 66-year-old female patient with frequent premature ventricular contractions (PVCs) came to our center with a history of palpitations for 1 year. She took β-blockers and propafenone, which had poor effects. She had no structural heart disease, coronary heart disease, or hypertension. The ECG and ambulatory ECG demonstrated monomorphic PVCs with the morphology of a right bundle branch block (RBBB) in the V1 lead, a positive QRS complex in the II, III, and aVF leads, and a “QS” pattern in the I lead, suggesting the origin of the left ventricular outflow tract (LVOT) (Fig. 3a). During the EPS, the earliest ventricular potential (VP), which was 27 ms preceding the onset of the QRS wave on the surface ECG, was detected in the left coronary cusp. RF ablation was performed via an ablation catheter (Thermocool SMART-TOUCH CF-sensing ablation catheter, Biosense Webster, Diamond Bar, Calif) with a setting of 35 W/50°C, and the PVCs decreased but did not disappear. Then, we moved the ablation catheter forward to the mitral annulus, but we obtained no earlier VP. The PVCs did not vanish when RF ablation was performed with a same setting at the high septum of the right ventricular outflow tract (RVOT), where the VP was 21 ms preceding the onset of the surface QRS wave. Then, we placed an RF catheter distal to the GCV and obtained a VP that was 21 ms preceding the onset of the QRS wave on the surface ECG (Fig. 3b, c). Coronary angiography and venography were performed to identify the GCV and ensure a safe proximity between it and the adjacent coronary artery. RF energy was applied in the GCV with a 56-hole irrigated-tip radiofrequency catheter (Thermocool Smarttouch SF; Biosense Webster, Diamond Bar, Calif) at a maximum power of 45 W, an irrigation rate of 30 mL/min and a temperature of 50°C for 5 s, and the PVCs vanished immediately. The total application time was 25 s, and no PVC occurred during the following 30 min. No coronary artery damage was confirmed after coronary angiography. No tachycardia occurred at the 15-month follow-up.

#### Case 4

**Fig. 4:**
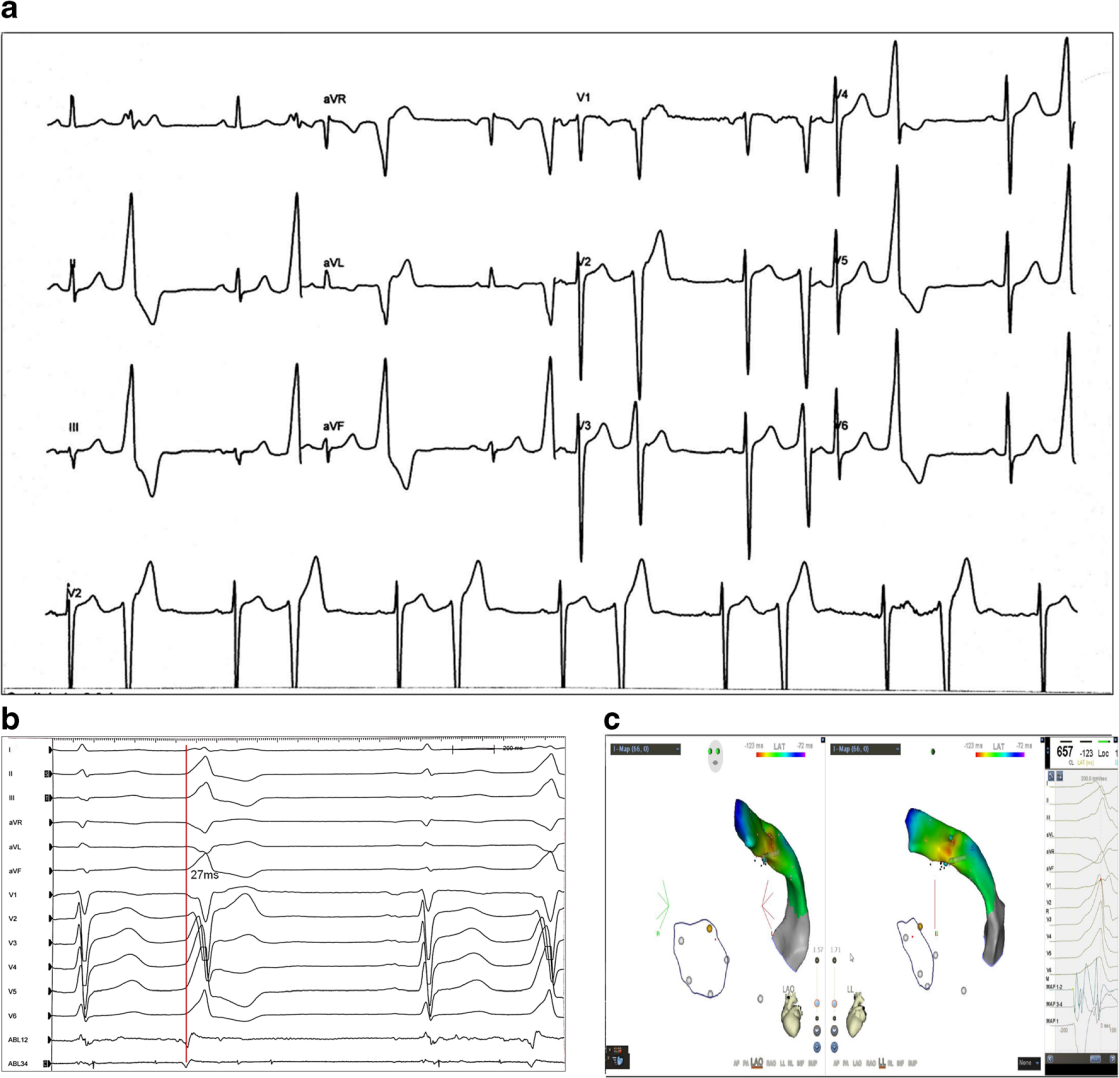
Case 4—surface ECG and intracardiac electrogram during EPS study**. a** The ECG demonstrated PVC. **b**, **c** Shows the ablation site

A 49-year-old man with frequent PVCs suffered from palpitations and shortness of breath for 2 months. He had no structural heart disease, coronary heart disease, or hypertension. The echocardiography was normal. The ambulatory ECG demonstrated monomorphic PVCs and non-sustained ventricular tachycardia (NSVT). The surface ECG showed PVCs with the morphology of a left bundle branch block (LBBB), a positive QRS complex in the II, III, and aVF leads, and an “rS” pattern in the I lead and late transition in the precordial lead, suggesting the origin of the RVOT (Fig. 4a). During the EPS, we mapped the earliest VP at the high septum of the RVOT, which was 31 ms preceding the onset of the QRS wave on the surface ECG. RF energy was applied at 35 W/50°C with a catheter (Thermocool SMART-TOUCH CF-sensing ablation catheter, Biosense Webster, Diamond Bar, Calif), and PVCs became less frequent but did not disappear. Then, we obtained a VP distal to the GCV, which was 27 ms preceding the onset of the QRS wave on the surface ECG (Fig. 4b, c). RF energy was administered at the site by a 56-hole irrigated-tip RF catheter (Thermocool Smarttouch SF; Biosense Webster, Diamond Bar, Calif), after confirming safe proximity, with a maximum power of 45 W, an irrigation rate of 30 mL/min and a temperature of 50 °C, and the PVCs vanished immediately. The total application time was 25 s, and no PVC occurred during the following 30 min. No coronary artery damage was detected on coronary angiography. No PVC or VT occurred at the 5-month follow-up.

### Animal study

#### Swine heart preparation

Four swine hearts (all males, weight 450 g±15 g, age 12–15 months) were prepared *in vitro*. The isolated heart model was based on the method described by Langendorff [1]. It consisted of a saline-filled container, including a thermostat to keep the temperature at 37 °C, a circulating pump, an indifferent electrode, and porcine heart preparations immersed in saline after pericardium excision. The heart was filled with heparinized swine blood with a fluid temperature of 37 °C at a perfusion pressure of 60–70 mmHg and flow velocity of 160 mL/min in the coronary sinus to simulate the characteristics of blood flow *in vivo* [2].

#### Ablation protocol

The procedure was carried out at the CSO and MCV. The initial impedance was 190–210 Ω, and the force range was 10–20 g. The titration RF ablation was performed under 45 W/5 S, 5 rounds, for a total duration of 25 s, in the study group (HPSD: 45 W/5×5 S). In the control group, titration ablation was performed under 25 W/10 S, 5 rounds, for a total duration of 50 s (LPLD: 25 W/5×10 S). RF ablation was performed with a 56-hole irrigated-tip RF catheter (Thermocool Smarttouch SF; Biosense Webster, Diamond Bar, Calif). The saline irrigation rate for the catheter tip was set at 30 mL/min for HPSD and at 25 mL/min for LPLD. The saline irrigation rate was maintained at 2 mL/min between applications. The application was terminated immediately if steam pop occurred.

#### Lesion examination

Lesion length was defined as the maximal longitudinal surface diameter, and lesion width was defined as the maximal horizontal surface diameter. The heart was cut along the maximal longitudinal surface diameter to measure lesion depth, and the following parameters were determined: maximal depth (D), maximal width (W), and maximal length (L). Because the lesions were made with the catheter in a position parallel to the tissue, the lesion volume was calculated with the formula for an ellipsoid as follows [3]:

$$ Lesion\ Volume=\left(\frac{4}{3}\pi \times D\times \frac{1}{2}W\times \frac{1}{2}L\right)\times \frac{1}{2}. $$

#### Statistical analysis

All analyses were performed using SPSS version 22.0 (SPSS Inc., Chicago, IL, USA). Continuous variables are expressed as the mean ± SD if normally distributed; the median and the 25 to 75% interquartile range were used if the data were clearly skewed. An unpaired *t* test or one-way analysis of variance was performed for measurement data. For categorical variables, chi-square tests or Fisher’s exact tests were used. A 2-tailed *P* value < 0.05 was considered statistically significant.

## Results

### RF ablation with HPSD and LPLD

**Fig. 5:**
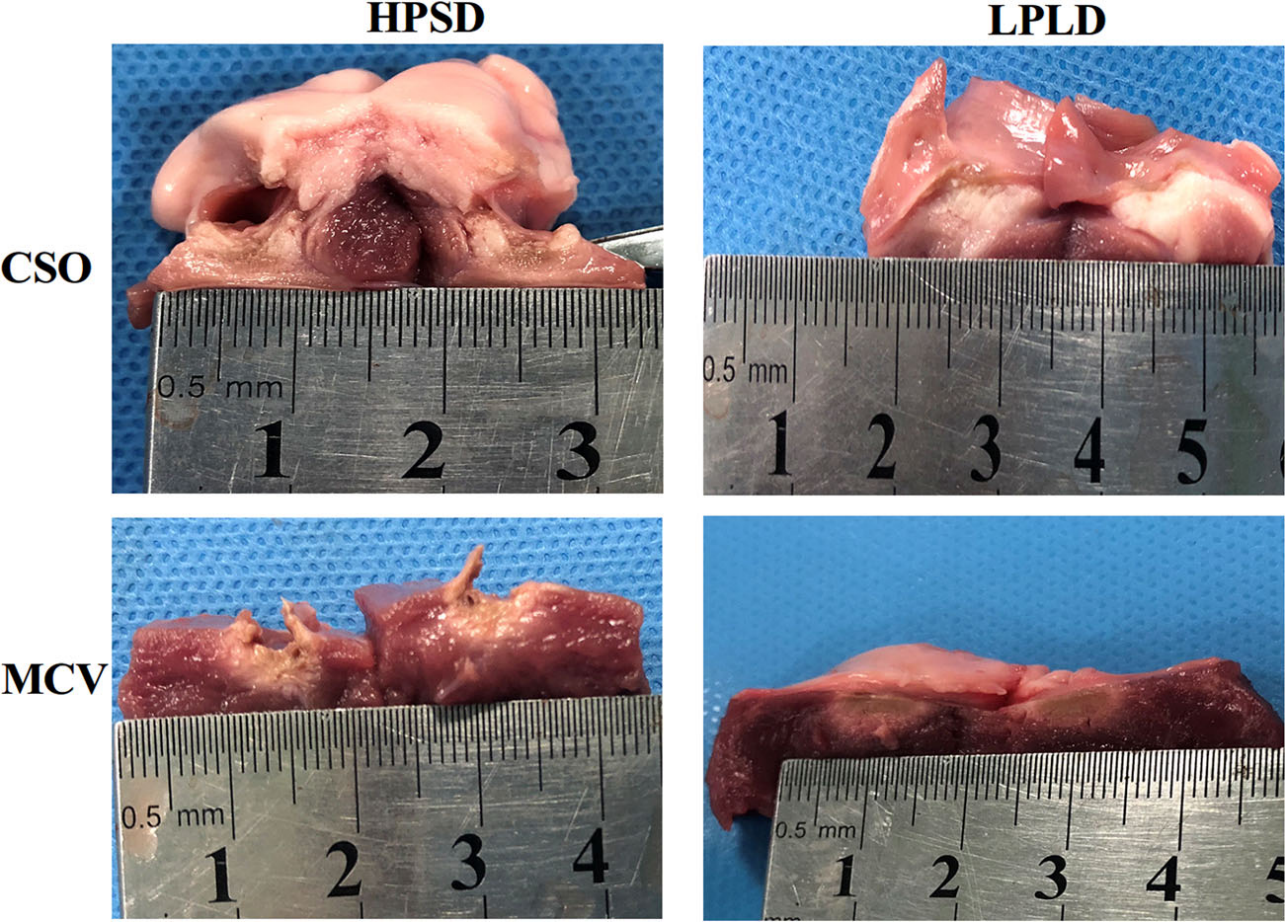
Gross appearance of swine heart tissue. (HPSD, high-power, short-duration; LPLD, low-power, long-duration; CSO, coronary sinus ostium; MCV, middle cardiac vein)

There were 28 lesions created in a total of 4 swine hearts, including 12 ablations produced in the CSO and 16 produced in the MCV (Fig. 5). HPSD lesions (45 W/25 S, *n*=16) and LPLD lesions (25 W/50 S, *n*=12) are compared in Table 1. HPSD lesions were longer, wider, deeper, and larger than LPLD lesions. However, there was a significant difference only in mean width (9.34±2.14 mm vs. 7.78±1.71 mm, *P*=0.041) between the 2 groups.

**Table 1:** Geometrical characteristics of HPSD lesions and LPLD lesions

	HPSD lesions (45 W/25 S, *n*=16)	LPLD lesions (25 W/50 S, =12)	*P*-value
Lesion length, mm	8.6±1.4	8.43±1.61	0.765
Lesion width, mm	9.34±2.14	7.78±1.71	0.041
Lesion depth, mm	4.66±1.12	4.29±0.99	0.375
Lesion volume, mm^3^	410.95±169.58	310.5±196.52	0.159

*HPSD* high-power, short-duration; *LPLD* low-power, long-duration

### RF ablation at the CSO

A total of 12 ablations were carried out in the CSO, 4 of which used the LPLD strategy, while the other 8 used the HPSD strategy. Lesion metrics are shown in Table 2, and no significant differences were found in geometric parameters between the 2 groups.

**Table 2:** Geometrical characteristics of HPSD lesions and LPLD lesions at CSO and MCV

	HPSD lesions (45 W/25 S)	LPLD lesions (25 W/50 S)	*P*-value
*CSO*, *n*	8	4	12
Lesion length, mm	8.72±1.76	8.36±1.87	0.748
Lesion width, mm	9.44±2.21	8.66±1.39	0.533
Lesion depth, mm	4.64±1.48	5.03±1.43	0.67
Lesion volume, mm^3^	428.54±210.11	424.93±314	0.981
Steam pop, n	0	0	0
*MCV, n*	8	8	16
Lesion length, mm	8.47±1.02	8.46±1.6	0.985
Lesion width, mm	9.38±2.23	7.34±1.76	0.065
Lesion depth, mm	4.68±0.75	3.299±0.42	0.026
Lesion volume, mm^3^	393.35±129.48	253.29±84.9	0.023
Steam pop, n	0	1	1

*HPSD* high-power, short-duration; *LPLD* low-power, long-duration; *CSO* coronary sinus ostium; *MCV* middle cardiac vein

### RF ablation at the MCV

A total of 16 ablations were carried out in the MCV, 4 of which were produced under the LPLD strategy, and another 8 were produced under the HPSD strategy. Lesion metrics are shown in Table 2. In brief, HPSD produced significantly deeper (4.68±0.75 mm vs. 3.299±0.42 mm, *P*=0.026) and larger (393.35±129.48 mm3 vs. 253.29±84.9 mm3, *P*=0.023) lesions than LPLD. One instance of steam pop occurred in the LPLD group.

## Discussion

In this study, we presented clinical application of HPSD in 4 patients with 3 different types of arrhythmias and observed the geometric characteristics of lesions created by HPSD in the coronary sinus in swine hearts. The main finds of this study show that HPSD produce longer, wider, deeper, and larger lesions than LPLD in coronary sinus; moreover, this difference was more pronounced when the ablation was in the MCV. HPSD has a reliable efficiency not only for PVI but also for ablation in the coronary sinus.

### HPSD

RF ablation lesions are generated by thermal injury: resistive heating and conductive heating. Resistive heating occurs on tissues immediately, and conductive heating occurs slowly but contributes more to lesion growth. Conductive heating may extend to deeper tissue layers and result in peripheral tissue damage. HPSD improves the relationship between resistive heating and conductive heating, allowing immediate heating to result in a larger, permanent tissue injury while restricting conductive heating. Felix Bourier et al. [4] compared lesion metrics of HPSD application and standard power application in an in silico simulation and a porcine *in vitro* model. HPSD can produce a larger and shallower lesion, and lesion metrics increase fastest during the first 10 s regardless of the power setting. Eran Leshem et al. [5] researched the characteristics of HPSD lesions in 20 beating swine hearts and found that HPSD produce a wider, transmural lesion with 100% contiguous lines, compared with standard power ablation, which yields smaller lesions with 25% linear gaps and 29% partial thickness. Our study compared the differences in geometric parameters of lesions between HPSD and LPLD in the coronary sinus. The HPSD lesions were larger and deeper than LPLD lesions, but the difference was significant only in the MCV. It may be due to the low blood flow in the coronary sinus and its different anatomical structures from the pulmonary vein.

Nilsson B et al. [6] compared the effectiveness of segmental PVI between HPSD and traditional application. HPSD reduced PVI time, mean fluoroscopy time with a similar outcome. The efficacy and safety of very high-power, short-duration ablation was also evaluated [7]. The procedure and fluoroscopy times were lower than those of conventional ablation, with no increased complications.

Castrejón-Castrejón et al. [8] found that asymptomatic esophageal lesions occur more frequently in conventional applications than in HPSD according to upper digestive tract endoscopy. The authors indicated that longer application time play more important role in esophageal damage than the power setting. HPSD did not increase the risk of esophageal thermal injury [9]. Winkle RA et al. [10] examined complication rates for 13974 of ablations at RF powers ranging from 45–50 W in 10,284 patients from 4 centers. Three atrial esophageal fistulas occurred in the lower-power group (35 W/20 s on the posterior wall), but only 1 atrial esophageal fistula occurred in the high-power group (45–50 W/2–10 s on the posterior wall). The incidence of audible steam pop, pericardial effusion, and esophageal lesions was extremely low in the HPSD group.

Although HPSD shows a comparable safety profile to conventional ablation, the safety margin of HPSD is very narrow. To predict lesion dimensions properly, some algorithms were created and have been proven to be effective and safe [11, 12]. It was shown that high-power ablation guided by the ablation index (AI) is safe and efficient [13, 14]. However, a variety of AI or lesion size index (LSI) values were set in these studies, and the explicit values are still in question, especially when the posterior wall is ablated. Unipolar signal modification may be another effective parameter for guiding high-power RF ablation for PVI [15]. However, it is only used in ablation for paroxysmal AF, and it cannot distinguish electrical stunning from cellular necrosis, which may result in false-positive transmural lesions, especially in conventional power ablation.

The acute outcome of HPSD has been proven, but its long-term outcome is still controversial [16]. Moreover, the specific reproducible ablation parameters and endpoint should be determined, and whether the power be reduced when ablating posterior wall is still a question.

### Electrophysiological characteristics of the coronary sinus

The coronary sinus is in the atrioventricular groove, and it is surrounded by myocardial sleeves, which are composed of bands of muscle from the left atrium (LA) and right atrium (RA), some of which have ectopic automaticity [17]. It was named the coronary sinus muscle extension (CSE), and these specific anatomical sites and adjacent pericardial tissues may be sources of arrhythmias such as AT, AVRT, PVC, and idiopathic VT. It is difficult to produce transmural, durable lesions at these sites because of its complex anatomy, the venous characteristics of its large lumen and thin wall, interrupted ablation due to increased impedance, and inadequate power delivery due to the effect of a “heat sink” caused by blood flow in the coronary sinus system [18]. The coronary sinus and its branches are close to the left circumflex artery and posterolateral branches of the right coronary artery; thus, it is critical to avoid damage to the vein and adjacent coronary artery during ablation. The strategy of lower power and a short duration and performing angiography before the procedure to determine anatomical proximity are useful to reduce the risk of coronary injuries [19, 20]. However, low power may lead to insufficient ablation, and a long duration may generate discontinuous energy delivery due to increased impedance, leading to extra damage to collateral tissue. Obtaining sufficient energy delivery and avoiding damage to adjacent tissue are critical to successful ablation in the coronary sinus [21].

### HPSD in the coronary sinus

As mentioned above, increasing the power setting properly with a short duration may be an alternative ablation protocol with a promising safety status. HPSD was applied to in GCV (maximum power 50 W, 30 s for each lesion) in a patient with idiopathic VT [22]. No arrhythmia occurred during the 8-month follow-up after the procedure.

The feasibility and safety of HPSD in the coronary sinus were also verified in our study. Titrated HPSD can produce lesions that are approximately similar in length, width, depth, and volume in a short time. The volume and depth of titrated HPSD lesions are larger than those of LPLD lesions. It is interesting that the difference is more significant in the MCV, which may be due to the weak effect of the “heat sink” because of poor blood flow in the MCV [18]. No “pop” occurred in HPSD.

We performed HPSD at the MCV, CSO, and GCV after a previous unsuccessful ablation with the traditional LPLD strategy in 4 patients, and a satisfactory outcome was achieved. RF ablation was performed by a 56-hole irrigated-tip RF catheter with an irrigation rate of 30 mL/min and a temperature of 50 °C. In consideration of the possible damage to coronary sinus with high power, each HPSD procedure only lasted for 5 s (45 W/5 S) and 5 rounds were performed. No coronary artery damage was confirmed according to the coronary angiography.

### Limitations

The study has several limitations. First, the animal study was an *in vitro* experiment not an *in vivo* experiment, and HPSD was carried out on non-beating hearts. The isolated heart model may not fully reflect real blood flow in the coronary sinus, influencing the experimental results. So, the data are not fully representative of lesions characteristics in patients. Second, the sample size of the animal research and the number of clinical cases are small, and there is a lack of histological observation of HPSD lesions. Four case reports are inadequate to demonstrate safety. More *in vivo* animal studies and clinical cases are needed. Third, the evaluation of complications and risk was only focused on coronary artery damage; however, we did not assess the risk of other complications, such as esophageal injury. Fourth, none of the 4 patients had structural heart disease or coronary heart disease, and whether HPSD can be applied to these patients remains unclear. Finally, the feasibility of HPSD in other arrhythmias, such as atrial flutter and VT, needs further study.

## Conclusion

Compared with conventional ablation procedures, HPSD in the coronary sinus is efficacious. Although the safety needs to be further evaluated based on more animal and clinical studies, HPSD is an alternative strategy for ablation in the coronary sinus.

## Data Availability

All the data supporting our findings are contained within the manuscript.
